# Exploring the Efficacy of Platinum and Palladium Nanostructures for Organic Molecule Detection via Raman Spectroscopy

**DOI:** 10.3390/s18010147

**Published:** 2018-01-07

**Authors:** Minh Tran, Alison Whale, Sonal Padalkar

**Affiliations:** 1 Department of Mechanical Engineering, Iowa State University, Ames, IA 50011, USA; mhtran@iastate.edu; 2 Department of Materials Science and Engineering, Iowa State University, Ames, IA 50011, USA; awhale@iastate.edu; 3 Microelectronics Research Center, Iowa State University, Ames, IA 50011, USA

**Keywords:** morphology, size, density, nanostructures

## Abstract

Noble transition metals, like palladium (Pd) and platinum (Pt), have been well-known for their excellent catalytic and electrochemical properties. However, they have been considered non-active for surface enhanced Raman spectroscopy (SERS). In this work, we explore the scattering contributions of Pd and Pt for the detection of organic molecules. The Pd and Pt nanostructures were synthesized on silicon substrate using a modified galvanic displacement method. The results show Pt nanoparticles and dendritic Pd nanostructures with controlled density and size. The influence of surfactants, including sodium dodecyl sulfate and cetyltrimethylammonium bromide, on the size and morphology of the nanostructures was investigated. The Pd and Pt nanostructures with a combination of large size and high density were then used to explore their applicability for the detection of 10^−5^ M Rhodamine 6G and 10^−2^ M paraoxon.

## 1. Introduction

Galvanic displacement is one of the electrochemical methods used to fabricate metals on semiconductor surfaces [1,2]. Galvanic displacement is simple, inexpensive and self-limiting. It is also substrate-selective, since metals can only be deposited on substrates, where electron exchange can occur. This selectivity also leads to conformity of the coating, regardless of the geometric complexity of the substrates [3,4]. Due to these attributes, there has been an increase in the use of galvanic displacement for nanofabrication, in which metals, having distinct morphologies, are distributed randomly or with defined patterns on Si substrates [5,6,7,8,9,10,11]. Likewise, galvanic displacement was also used to deposit metals on 1D Si nanostructures [12,13,14,15]. Further, the growth and interface characteristics of metals on underlying semiconductor substrates by galvanic displacement have served as important topics for fundamental studies [14,16,17,18,19]. So far, a variety of metals has been deposited by galvanic displacement on Si and Ge substrates, including the noble metals, Au/Si [3,6,7,10,12,14,16,17,18,19,20,21,22], Au/Ge [3,8], Ag/Si [9,13,16,17,21,23], platinum-group metals, such as Pt/Si [7,11,16,24], Pt/Ge [8], Pd/Ge [8,16], Pd/Si [16], Rh/Si [16], base metals, such as Cu/Si [5,16,25,26,27,28,29,30,31,32], Ni/Si [27], and several metals on III-V semiconductor substrates [1].

In galvanic displacement, the semiconductor substrate acts as an electron source via its surface oxidation. Likewise, metal ions in solution, taking up electrons supplied by the substrate, are reduced to metal atoms. Thus, the overall displacement process occurs when metals are deposited on the semiconductor substrate and the surface oxide is dissolved, as described by the following chemical equations [1,3]:

(1) $$\mathrm{Anodic}:\;\mathrm{Si}\;\left(s\right)+6F^{-}\;\left(\mathrm{aq}\right)\to {\mathrm{SiF}}_{6}^{2-}\;\left(\mathrm{aq}\right)+4e^{-}$$

(2) $$\mathrm{Cathodic}:\;M^{n+}\;\left(\mathrm{aq}\right)+{\mathrm{ne}}^{-}\to M\;\left(s\right)$$

(3) $$\mathrm{Overall}:\;M^{n+}\;\left(\mathrm{aq}\right)+\mathrm{Si}\;\left(s\right)+6F^{-}\;\left(\mathrm{aq}\right)\to M\;\left(s\right)+{\mathrm{SiF}}_{6}^{2-}\;\left(\mathrm{aq}\right)$$

where M denotes any metal whose standard redox potential is higher than that of hydrogen. The role of hydrofluoric acid (HF) is to maintain the electron supply by dissolving the surface oxide.

Platinum (Pt) and palladium (Pd) are noble transition metals, whose excellent catalytic and electrochemical properties have been widely used in modern technologies, including chemical and biosensing [33,34,35], photocatalysis [36,37], electrocatalysis [38,39,40,41], and Si nanowire growth [4,42]. Although they are promising candidates for a variety of applications, they are not favorably suited for detection via Raman spectroscopy, also called as Surface Enhanced Raman Spectroscopy (SERS). These transition metals have interband excitation occurring in the visible light region and thus quenching the effect of surface plasmon resonance (SPR) [43,44,45], which has been considered the major mechanism behind the Raman signal enhancement observed in SERS-active materials, like Au and Ag. One method to improve the SERS efficiency of Pt and Pd is called borrowing SERS activity, in which the SERS substrate is comprised of a SERS-active core (Au or Ag) and a transition metal shell [46]. However, to avoid blocking the SPR effect of the rough core underneath, the transition metal shell is required to be atomically thin. Further, the shell must be pinhole free, to avoid the core from adsorbing the analytes. Both requirements can be challenging to fabricate [43,44]. Alternatively, Pt or Pd alone can be used as substrate material, whose surface nanostructure can be engineered to generate a SERS signal. Electrochemically roughened Pd and Pt surfaces were used to detect 0.01 M pyridine [47,48] and 10^−5^ M thiocyanate [49] with an enhancement factor (EF) up to three orders of magnitude for Pd, via SERS [48]. While the authors showed that SERS activities were dependent on surface roughness, the surface morphologies were often inhomogeneous across the surface and between substrates, leading to deviations in SERS measurements. Bartlett et al. reported that close packed hexagonal arrays of uniform and well-defined Pd and Pt nanovoids exhibited SERS spectra for 10^−2^ M benzenethiol, with significant EFs for Pd and Pt [45]. Monodisperse Pt and Pd nanostructures of several different morphologies have also been synthesized for SERS, including Pd urchins/flowers/hemispheres and Pt pinecones/microspheres/flowers by galvanic displacement for the detection of Rhodamine 6G (R6G) [38,50], Pd flowers/pinecones and Pt nanothorns by electrodeposition for 4-mercaptopyridine and pyridine [51,52] and Pd nanodendrites/polyhedra by solution-based chemical reduction for R6G and 4-mercaptobenzoic acid [41,53]. So far, analyte concentration as low as 10^−6^ M R6G and EF up to 10^5^ for 4-mercaptopyridine have been achieved with Pd urchins [50] and Pd flowers [51], respectively, which are comparable to those of Ag or Au.

Few authors have reported successful fabrication of metallic nanostructures on Si without HF [7,20]. As demonstrated in the present work, with a very short reaction time, the fluoride-free method, is likely to result in controlled density, size, and growth. Here, we report on the deposition of Pt and Pd on n-Si (100) substrate, using a modification of the galvanic displacement process. A very small amount of HF was added to the plating solution, and HF was concentrated at the solid–liquid interface, making metallic deposition more efficient. By using cyclic deposition, the size and morphology evolution was observed. Further, high density nanostructures were successfully achieved in the absence of concentrated reagents. The deposited Pd and Pt nanostructures were then used for SERS detection of R6G and paraoxon, with detection limits of 10^−5^ M and 10^−2^ M, as well as EFs up to 10^2^ and 10, respectively. Additionally, the influence of surfactants, including sodium dodecyl sulfate (SDS) and cetyltrimethylammonium bromide (CTAB), on the size and morphology of the nanostructures was investigated.

A range of characterization techniques were employed to assist the investigation. Scanning electron microscopy (SEM) was used to determine morphology, size, and density of the nanostructures. The chemical information of the nanostructures was obtained by energy dispersive X-ray spectroscopy (EDX). Finally, surface plasmon resonance (SPR) absorption and SERS properties of the nanostructures were recorded by UV-Vis spectroscopy and Raman spectroscopy, respectively.

## 2. Materials and Methods

### 2.1. Materials

The chemicals used for metallic deposition included potassium tetrachloroplatinate(II) (K_2_PtCl_4_, ≥99.9%), sodium tetrachloropalladate(II) (Na_2_PdCl_4_, 98%), and cetyltrimethylammonium bromide (C_19_H_42_BrN, ≥99.9%) purchased from Sigma–Aldrich (Milwaukee, WI, USA), sodium dodecyl sulfate (C_12_H_25_NaO_4_S, ≥99%) from Fisher Scientific (Hampton, NH, USA), which were used as received. Hydrofluoric acid (HF, 48–51%) was purchased from Thermo Fisher Scientific (Waltham, MA, USA). For SERS experiments, Rhodamine 6G dye (R6G, 99%) and paraoxon-ethyl (C_10_H_14_NO_6_P, ≥90%) were purchased from Sigma–Aldrich (Milwaukee, WI, USA). All solutions were prepared using deionized (DI) water. HF was contained in a polypropylene beaker for experimental use. The substrate was Si (100) wafer (4″, single-crystalline, n-type, ρ = 3–9 Ωcm) purchased from El-Cat Inc. (Ridgefield Park, NJ, USA). Prior to deposition, Si wafer was cleaved into 1.5 cm × 1.5 cm^2^ pieces. The chemicals used for cleaning Si substrate were acetone and ethanol (100%, 200 proof).

### 2.2. Synthesis of Palladium and Platinum Nanostructures via Galvanic Displacement Method

The Pd and Pt nanostructures were synthesized by the galvanic displacement method. The synthesis procedure includes cleaning the Si substrate with acetone, followed by ethanol and DI water. Each cleaning step was performed for 10 min, in an ultrasonic bath, to degrease and de-contaminate the substrate. For the deposition, the Si substrate was immersed in 10% (*w*/*w*) HF solution for 2 min to eliminate surface oxide and activate the surface. The substrate was then instantly immersed for 5 min in 0.3 mM K_2_PtCl_4_ or Na_2_PdCl_4_ for Pt or Pd deposition, respectively, followed by rinsing with deionized water. This deposition procedure will be denoted as 1 deposition cycle, which includes immersion in HF, treating with the metal precursor solution and rinsing with DI water. Multiple deposition cycles were also used in the present investigation. For experiments that were used to study the influence of surfactants on the final outcome, the metallic precursor solution was composed of 0.1 mM SDS or CTAB with 0.3 mM K_2_PtCl_4_ or Na_2_PdCl_4_. Finally, the Pt and Pd nanostructures were air dried and stored for further characterization.

The R6G dye solution was prepared by dissolving 0.8 mg of R6G in 5 mL of DI water and used as stock solution. Similarly, paraoxon stock solution was prepared by mixing 30 μL paraoxon with 0.98 mL DI water. The SERS substrates were prepared by using Si substrates with Pt or Pd nanostructures, on which a small volume (300 μL) of the stock solution was drop casted and dried under ambient conditions. Prior to drop casting the analyte, the sample was treated with ambient air plasma for 1 min. under medium radio frequency power level (11 W) by a plasma cleaner (PDC-001, Harrick Plasma, Ithaca, NY, USA).

### 2.3. Characterization

The size and morphology of the nanostructures were studied by scanning electron microscopy (SEM) using a FEI Quanta-250 (FEI, Hillsboro, OR, USA) SEM instrument at 10 kV accelerating voltage. The SEM instrument was equipped with an Oxford Aztec energy dispersive X-ray (EDX) analysis system, which was used to conduct elemental analyses of the deposited nanostructures. UV-Vis absorption spectroscopy was recorded by a Perkin Elmer Lambda 25 (Perkin Elmer, Waltham, MA, USA) spectrophotometer. To prepare samples for UV-Vis measurements, each sample was immersed in 1 mL DI water and sonicated at the highest power for 1–3 min to detach the metallic nanostructures from the Si substrate and disperse them in DI water. SERS measurements were performed at room temperature on a Renishaw Dispersive Raman Spectrometer (Renishaw, Wotton-under-Edge, UK) with an Ar-ion laser with a wavelength of 488 nm, using 50× objective lens, with incident power of 5 mW for 4 accumulations, each of 30 s duration. SERS spectra were collected from several random regions on each sample to confirm homogeneity.

## 3. Results and Discussions

### 3.1. Characterization of Palladium and Platinum Nanostructures

**Figure 1:**
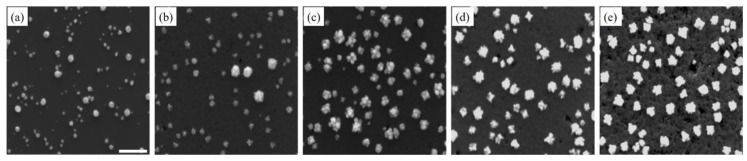
The Pd nanostructures are seen in SEM images. These images were taken at different stages of the deposition process, beginning with the completion of the first deposition cycle (**a**), followed by the third (**b**), fifth (**c**), eighth (**d**) and tenth (**e**) deposition cycles. The scale bar is 500 nm.

Figure 1 correlates the number of deposition cycles with the density, morphology evolution, and size of Pd nanostructures on the Si substrate (additional low and high magnification SEM images as well as size distribution and density charts can be seen in the Supplementary Materials).

Upon the completion of one deposition cycle, Pd nanostructures appeared to be quasi-spherical (Figure 1a). The large size distribution indicated that nucleation and growth occurred simultaneously, following the progressive nucleation mode. When the number of deposition cycles increased, Pd nanostructures also increased in size (Figure 1b,c). The growth was anisotropic, due to new Pd atoms being preferably deposited on high-energy facets in the radial direction. Pd nanostructures, thus, had very rough surfaces. In several cases, the anisotropic growth led to the formation of flower-like structures or clusters of small Pd nanostructures. With the increase in the deposition cycles, the dendritic Pd nanostructures appeared more compact, minimizing the surface area to achieve a stable configuration (Figure 1d,e) [54]. Likewise, when the number of deposition cycles increased, the size distribution was reduced. After repeated deposition cycles, the native oxide on the Si substrate was continuously dissolved by HF, creating a very rough and porous Si layer on the substrate. This layer had high electrical resistance, enough to hinder electron transfer between Pd and the substrate, and suppresses nucleation. In addition, more Pd nanostructures acted as nucleation centers to compete with the Si substrate for new Pd atoms. New Pd atoms would preferably adsorb on the existing Pd nanostructures, since they had higher electronegativity than Si [42].

**Figure 2:**
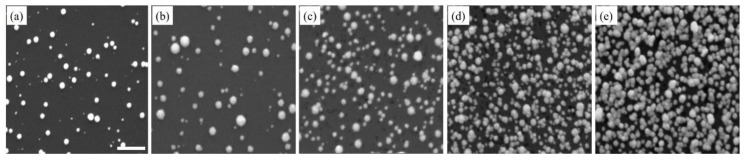
The Pt nanostructures are seen in SEM images. These images were taken at different stages of the deposition process, beginning with the completion of the first deposition cycle (**a**), followed by the third (**b**), fifth (**c**), eighth (**d**) and tenth (**e**) deposition cycles. The scale bar is 500 nm.

In contrast, Pt nanostructures showed a steady increase in density, with respect to an increasing number of deposition cycles (Figure 2). This increase in density was a result of the continuous formation of new nuclei at every deposition cycle, as the growth and nucleation followed the progressive mode. However, it is also noteworthy that Pt nanostructures had more uniform morphology and relatively smooth surfaces with repeated deposition cycles (Figures S3, S4, S5b). In addition, while Pt nanostructures grew larger in size, their growth was isotropic and they maintained a spherical shape. When Pt nanostructures were sufficiently close to each other, they diffused and aggregated. At high densities, fusion between Pt nanostructures occurred, resulting in large regions of continuous Pt up to a micron in size. In Figure 1 and Figure 2, a considerable number of deep pits of different sizes were noticed on the substrate surface. On the n-type Si substrate, these pits were the locations of surface defects, and electron/hole exchange occurred locally at these pits during the very early stages of deposition [55,56].

**Figure 3:**
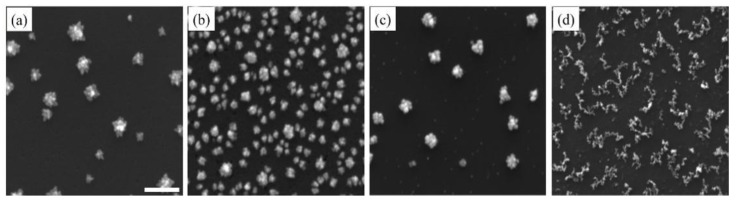
SEM images of Pd nanostructures deposited on Si substrate after the (**a**) first and (**b**) tenth deposition cycles in the presence of sodium dodecyl sulfate (SDS) surfactant, and (**c**) first and (**d**) tenth deposition cycles with cetyltrimethylammonium bromide (CTAB) surfactant. The scale bar is 500 nm.

Sodium dodecyl sulfate (SDS), an anionic surfactant, and cetyltrimethylammonium bromide (CTAB), a cationic surfactant, have been widely used as stabilizers [57,58] and morphology-directing reagents [50,59,60] for the growth of various metallic nanostructures. As shown in Figure 3, upon the addition of SDS and CTAB, dendritic Pd nanostructures were observed after just one deposition cycle (Figure 3a,c). Clearly, the presence of surfactants promoted anisotropic growth by selectively adsorbing on certain crystal facets of the growing Pd nanostructures and slowing down the diffusion of Pd atoms to those facets. The dendritic Pd nanostructures with SDS also had longer and sharper branches, indicating that SDS had a greater influence on the anisotropic growth. Furthermore, adding either SDS or CTAB appeared to accelerate the growth of Pd nanostructures. In the case of SDS, the adsorption of free anionic surfactant molecules to the surfaces would have given Pd nanostructures additional negative charges, which electrostatically attracted Pd atoms toward them. On the other hand, at the concentration of 0.1 mM, CTAB would adsorb on the surfaces of the Pd nanostructures as both sub-micelle aggregates and free surfactant molecules [61]. Their positively charged head groups, electrostatically binding PdCl_4_^2−^, thus attract and concentrate Pd precursors toward the Pd nanostructures. Figure 3b,d shows Pd nanostructures with SDS and CTAB after ten deposition cycles, respectively. The Pd nanostructures with SDS were smaller when compared with Pd nanostructures without surfactant. Their branches were also shorter and blunt after ten deposition cycles. The observed shrinking of Pd nanostructures after multiple deposition cycles could be a combination of the increasing impeded growth caused by a denser and thicker surfactant layer, and the rearrangement of the high-energy and unstable Pd atoms at the sharp tips of the branches. On the other hand, Pd nanostructures with CTAB, after ten deposition cycles, transformed into 2D fragmented networks of much smaller Pd nanoparticles. The mechanism behind the formation of such structures is unclear and should be subject to further investigation. Additionally, these nanostructures will be investigated for the detection of R6G in future studies.

**Figure 4:**
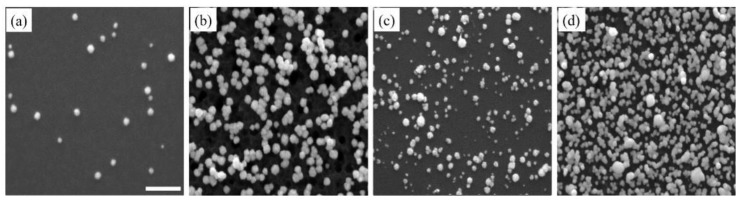
SEM images of Pt nanostructures deposited on Si substrate after the (**a**) first and (**b**) tenth deposition cycles in the presence of SDS surfactant, and (**c**) first and (**d**) tenth deposition cycles with CTAB surfactant. The scale bar is 500 nm.

**Figure 5:**
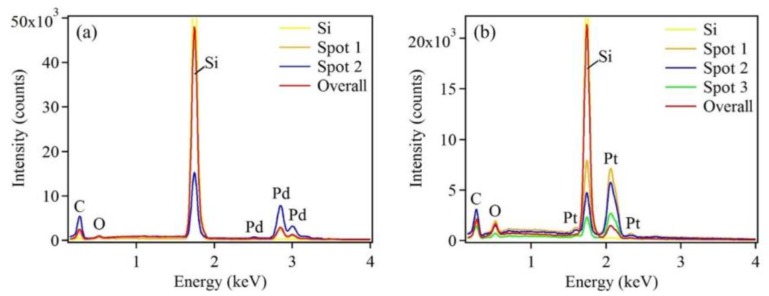
EDX patterns of (**a**) Pd and (**b**) Pt nanostructures deposited on Si substrates after five deposition cycles, showing the presence of Pd and Pt, respectively. EDX patterns recorded from individual nanostructures are presented as spots 1–3.

Figure 4 shows SEM images of Pt nanostructures, obtained by the addition of surfactants, like SDS and CTAB. Unlike the Pd nanostructures, there was no noticeable difference between Pt nanostructures, with and without SDS, indicating that SDS had limited influence toward the growth of Pt nanostructures (Figure 4a,b). In Figure 4b there was a large number of pits on the Si surface, which could be due to Pt nanostructure detachment. The addition of CTAB considerably increased the density of Pt nanostructures. The formation of sub-micelle aggregates facilitated the galvanic displacement process by confining metal ions close to the surface of the sub-micelles, thus increasing the reaction rate by bringing the reactants closer together at the micellar interface [61]. The morphology, however, slightly deviated from the spherical shape and this can be attributed to the slight anisotropic growth induced by selective adsorption of CTAB (Figure 4c). With further deposition cycles (Figure 5d), a high density of Pt nanostructures was observed. The densely-packed nanostructures combined with anisotropic growth led to coalescence of nearby nanostructures and a sub-monolayer coverage.

Chemical compositions of the as-synthesized samples were determined by EDX (Figure 5). The Pd or Pt peaks were observed for each respective metal-deposited sample. In addition, different Pd or Pt peak intensities were observed at different spots, reflecting the different sizes of the nanostructures. A very strong Si peak was obtained from the blank silicon substrate and from the sample substrate. The C peak was detected due to organic contaminants adsorbing on the sample surface, prior to its introduction to the SEM vacuum chamber, while the O peak was a result of the slight oxidation of the Si surface.

**Figure 6:**
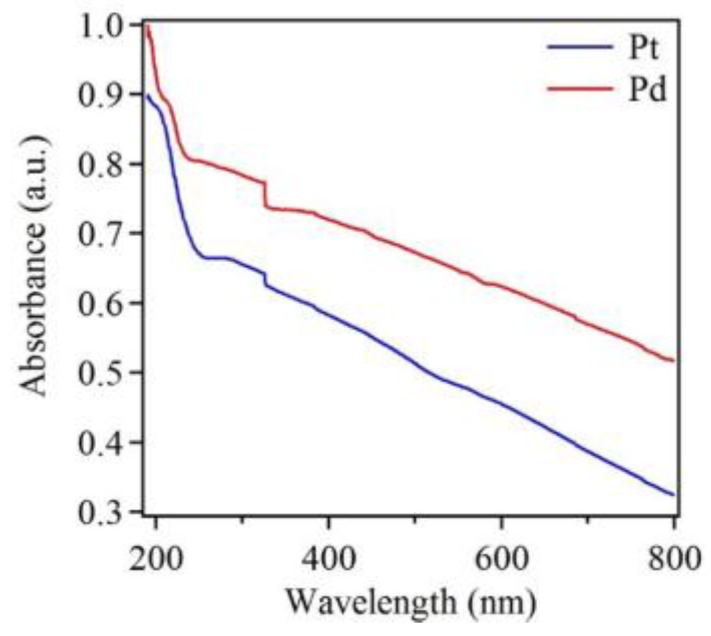
Normalized UV-Vis spectra of Pt and Pd nanostructures deposited on Si substrates after five deposition cycles, showing characteristic peaks and increasing absorption toward shorter wavelengths. The spectrum of Pt nanostructures is shifted downward for clarity.

In addition to EDX, UV-Vis data was also obtained. Figure 6 shows UV-Vis spectra of Pt and Pd nanostructures deposited on Si substrates. Both spectra showed no noticeable absorption peak in the visible region, and both showed increasing absorption toward the ultraviolet region. This result thus provided evidence that Pt and Pd did not have SPR properties in the visible region, as mentioned above. In addition, both the spectra show two small and broad absorption bands, centered at 210 nm and 325 nm for Pd, as well as at 194 nm and 313 nm for Pt. The UV-Vis absorption data were also in agreement with those reported in the literature [62,63]. Additionally, there was a sharp change in absorption at 310 nm and this is attributed to instrumental artifact. The UV-Vis absorption spectrum for blank Si substrate is shown in the Supplementary Materials (Figure S8), which matches well with the literature [64]. Additionally, transmittance data of Pt, Pd and blank Si is presented in the Supplementary Materials (Figure S9).

### 3.2. Palladium and Platinum Nanostructures as SERS Platforms

**Figure 7:**
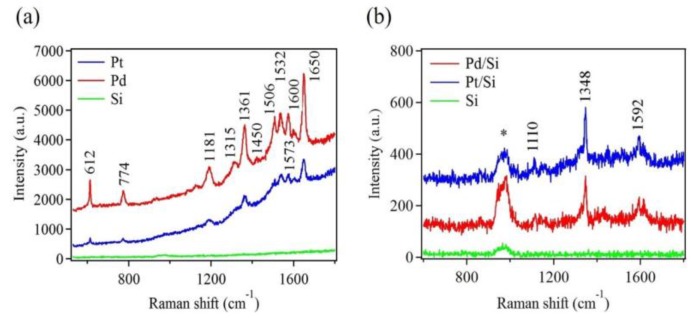
Raman spectra of (**a**) 10^−5^ M Rhodamine 6G (R6G) and (**b**) 10^−2^ M paraoxon adsorbed on Pd and Pt nanostructures deposited on Si substrates after ten deposition cycles. Raman spectra of R6G and paraoxon adsorbed on bare Si substrates are shown for comparison. The (*) indicates the Raman mode of Si.

To evaluate the Pt and Pd nanostructures as possible platforms for SERS application, R6G was chosen as a probe molecule. R6G has been widely used for SERS due to their well-defined vibrational features. To obtain the best performance of SERS substrates, samples with a combination of high-density and large metallic nanostructures were used. These samples were achieved after ten deposition cycles (see Figure S5 and Table S1). Figure 7a shows Raman spectra of 10^−5^ M R6G dye, drop casted onto Pt and Pd nanostructures. For comparison, the same concentration of R6G was also drop cast onto bare Si substrate without metallic deposition. No noticeable Raman signal was detected using this bare Si substrate. On the other hand, several strong Raman modes were observed for the Pd nanostructure sample. Nearly all of these Raman modes were also visible for the Pt nanostructure sample. However, their intensities were significantly reduced.

In addition to R6G detection, paraoxon was used, which served as a real sample. Here, Pt and Pd nanostructure platforms were used to demonstrate their ability to detect a real sample like paraoxon. Paraoxon is an organophosphorus compound and is used to regulate pests in crops and plants. It is highly toxic, causing detrimental health problems if exposed to food and drinking water [65]. SERS can be used for rapid detection of such pesticides. Figure 7b shows Raman spectra of a 10^−2^ M paraoxon drop casted on Pt and Pd nanostructures. The three strongest fingerprint peaks, representing different vibrational modes of the paraoxon molecule, were clearly seen in these spectra [66]. Raman scattering was certainly enhanced by the metallic nanostructures because no other peaks, except that of Si, were observed when bare Si was used as a SERS substrate. Table 1 provides a detailed assignment for all the Raman modes in Figure 7, according to published data.

The enhancement factor (EF) was determined to quantitatively evaluate the effectiveness of the SERS substrate. The most widely used definition of the EF is described as

$$\mathrm{EF}=\frac{I_{\mathrm{SERS}}/c_{\mathrm{SERS}}}{I_{\mathrm{RS}}/c_{\mathrm{RS}}}$$

where I_SERS_ and I_RS_ are Raman intensities of SERS and non-SERS substrates, respectively, while c_SERS_ and c_RS_ are analyte concentrations used for SERS and non-SERS substrates, respectively [72]. In the present study, the same analyte concentrations were used for both substrates. Thus, EF was simply a ratio of I_SERS_ to I_RS_. However, since the Raman signal obtained from bare Si substrate (I_RS_) was too weak to be detected by our instrument, it was not possible to obtain absolute values for EF. Instead, we roughly estimated EFs in a manner similar to those reported by Yamamoto et al. [70]. Thus, the EF value for R6G on Pd was ~10^2^, which was ~three times higher than that for Pt. Similarly, EF values for paraoxon on Pd and Pt both were ~10.

The Raman enhancement can be attributed to the local electromagnetic enhancement [73,74]. For nanostructures of noble metals, the extent of electromagnetic enhancement depends not only on their material, but also on their morphology, size, and density. It has been reported that large electromagnetic enhancement occurs at sharp features or high curvature sites, due to the lightning-rod effect [51,73,75]. Thus, the Pd nanostructures would amplify the electromagnetic field surrounding them, and would be a promising SERS substrate. Tian et al. used the 3D finite difference time domain method to predict that the maximum enhancement would occur at the tip apex of the Pd nanostructure’s branch [75]. In addition, large field enhancement would also occur at the gaps between adjacent branches, called hot spots, due to the coupling effects between the branches. This morphological advantage was the key factor that increased the SERS performance of Pd nanostructures for R6G over that of Pt nanospheres. Furthermore, overall, Pd nanostructures were larger than Pt nanostructures (see the Supplementary Materials), and a larger size, in the case of transition metals, might lead to stronger enhancement [76]. Further improvement in SERS detection can be achieved from the Pd-deposited substrate by increasing the density of Pd nanostructures, making very small gaps between the nanostructures to generate hot spots [77]. Additionally, the surface roughness of the underlying Si substrate would also contribute to the enhanced Raman signal during the detection event. Further, a similar explanation will also hold for Pt nanostructures.

## 4. Conclusion

In summary, we report a modification of the galvanic displacement method, in which Pt and Pd nanostructures were deposited on an n-type Si substrate with limited exposure to HF. By performing multiple cycles of the metallic deposition steps, large dendritic Pd and Pt nanostructures with a high density and small size distribution were obtained. Further, the Pd and Pt nanostructures were successfully used as SERS platforms for detecting low concentrations of organic analytes. From the results, it is clearly evident that Pd and Pt nanostructures, prepared by the modified galvanic displacement method, can produce stable and reproducible nanostructures that can be used for SERS application. Additionally, SDS and CTAB surfactants were utilized, which produced an increase in anisotropic growth of the dendritic Pd nanostructures. A higher density of Pt nanostructures was observed when CTAB was utilized. Future investigation will mainly focus on Pt and Pd samples, prepared in the presence of surfactants.

## Figures and Tables

**Table 1:** Raman mode assignments for R6G and paraoxon, corresponding to Figure 7.

**Raman (cm^−1^)**	**Assignment for R6G**	**References**
612	In-plane bending of C–C–C ring	[67,68,69]
774	C–H out-of-plane bending	[67,68,69,70]
1181	In-plane xanthene ring deformation, C–H bending, N–H bending	[67,68,69,70]
1315	Aromatic C–C stretching	[67,68]
1361, 1450	Aromatic C–C stretching, in-plane C–H bending	[67,68,69,70]
1506, 1532	Aromatic C–C and C–N stretching, C–H and N–H bending	[67,68,70]
1573, 1600	Aromatic C–C stretching, in-plane N–H bending	[67,68,70]
1650	Aromatic C–C stretching, in-plane C–H bending	[67,68,69,70]
**Raman (cm^−1^)**	**Assignment for Paraoxon**	
1110	C–H band (in plane)/NO_2_ asymmetric stretching	[66,71]
1348	Symmetry stretching NO_2_	[66,71]
1592	Phenyl ring vibration	[66,71]

## Supplementary Materials

The following are available online at http://www.mdpi.com/1424-8220/18/1/147/s1.
