# Supplementary material for: Exploring the Efficacy of Platinum and Palladium Nanostructures for Organic Molecule Detection via Raman Spectroscopy
Source: Sensors (Basel). 2018 Jan 7;18(1):147. doi: 10.3390/s18010147 (PMC5796314; doi:10.3390/s18010147)
Supplement: Supplementary file 1 [file sensors-18-00147-s001.pdf]

Exploring the Efficacy of Platinum and Palladium Nanostructures for Organic Molecule Detection via Raman Spectroscopy

Supplementary Information

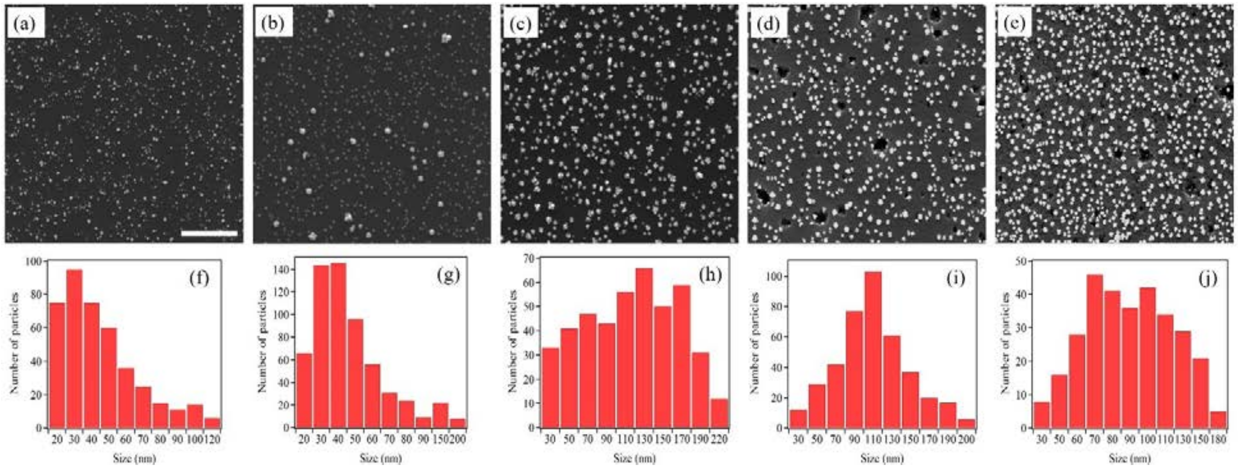

Figure S1. SEM images of Pd nanostructures deposited on Si substrate after (a) first, (b) third, (c) fifth, (d) eighth, and (e) tenth deposition cycle. The scale bar is 2 μm**.** (f-j) Size distributions of the corresponding Pd nanostructures.

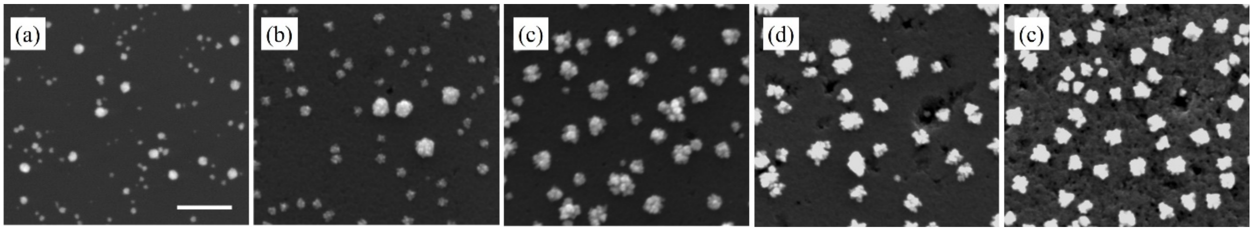

Figure S2. SEM images, at higher magnification, of Pd nanostructures deposited on Si substrate after (a) first, (b) third, (c) fifth, (d) eighth, and (e) tenth deposition cycle. The scale bar is 500 nm**.**

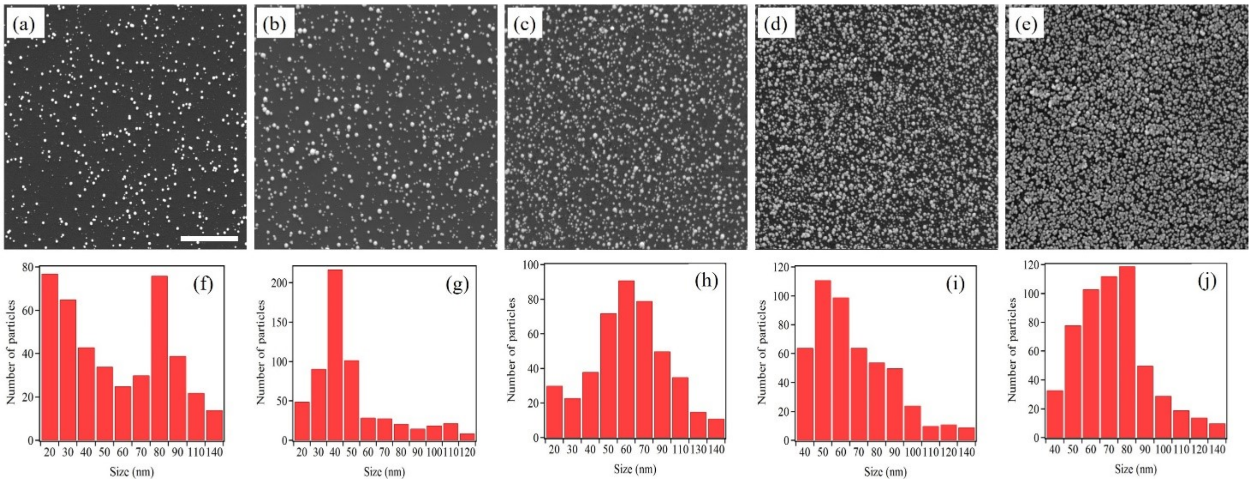

Figure S3. SEM images of Pt nanostructures deposited on Si substrate after (a) first, (b) third, (c) fifth, (d) eighth, and (e) tenth deposition cycle. The scale bar is 2 μm**.** (f-j) Size distributions of the corresponding Pt nanostructures.

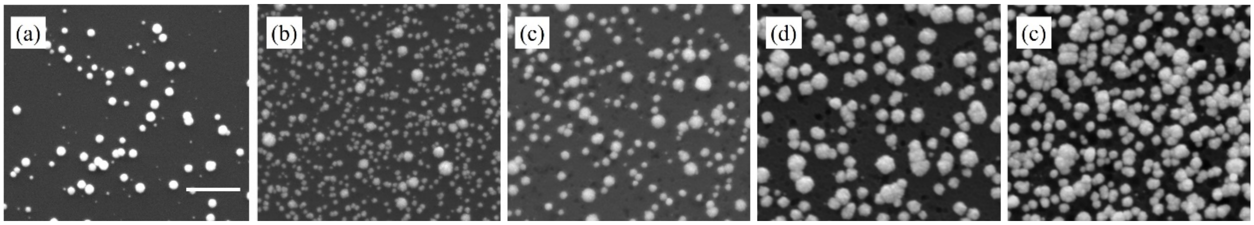

Figure S4: SEM images, at higher magnification, of Pt nanostructures deposited on Si substrate after (a) first, (b) third, (c) fifth, (d) eighth, and (e) tenth deposition cycle. The scale bar is 500 nm**.**

Table S1. Average sizes of Pd and Pt nanostructures after multiple deposition cycles

	One cycle	Three cycles	Five cycles	Eight cycles	Ten cycles
Pd	40 ± 22 nm	43 ± 27 nm	108 ± 51 nm	101 ± 39 nm	86 ± 29 nm
Pt	52 ± 29 nm	46 ± 27 nm	62 ± 30 nm	62 ± 22 nm	68 ± 21 nm

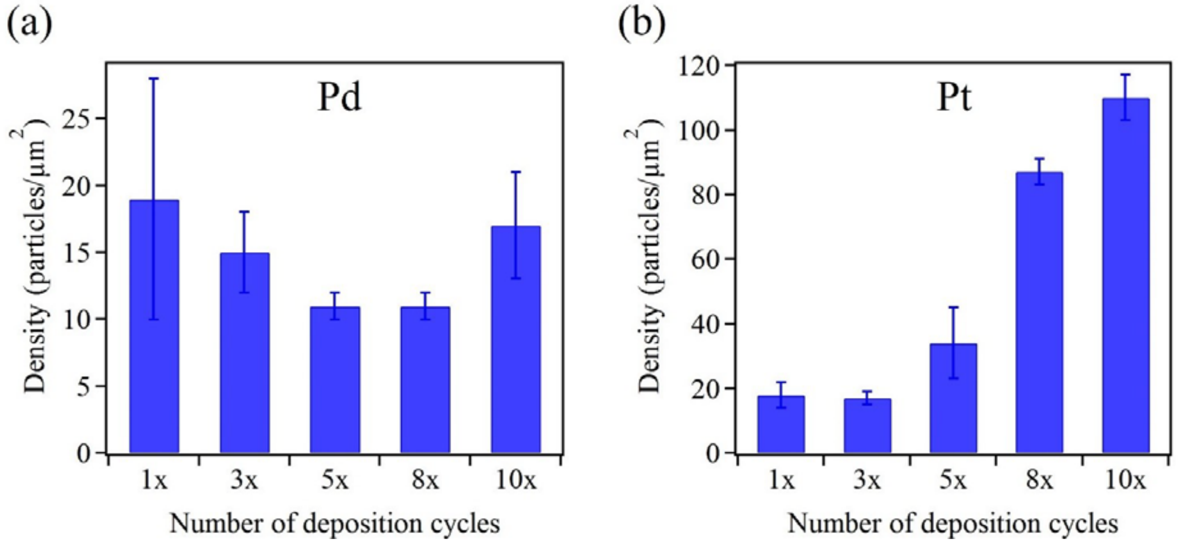

Figure S5. Number densities of (a) Pd and (b) Pt nanostructures after multiple deposition cycles. 1x-10x denote one to ten deposition cycles, respectively

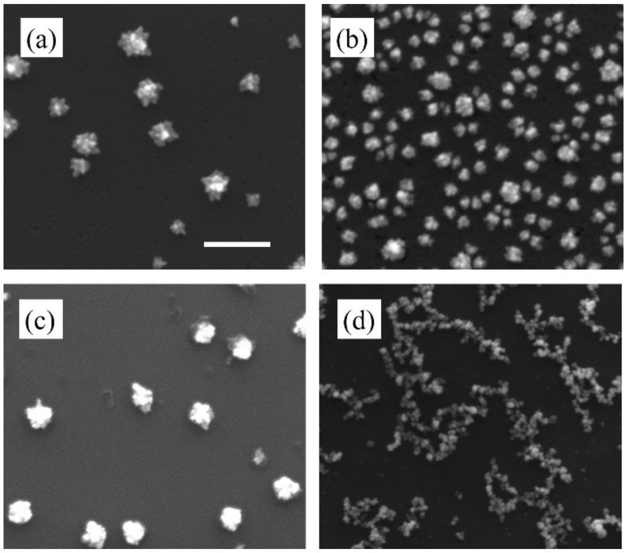

Figure S6. SEM images of Pd nanostructures deposited on Si substrate after (a) first, (b) tenth deposition cycle with SDS surfactant added, and (c) first, (d) tenth deposition cycle with CTAB surfactant added. The scale bar is 500 nm.

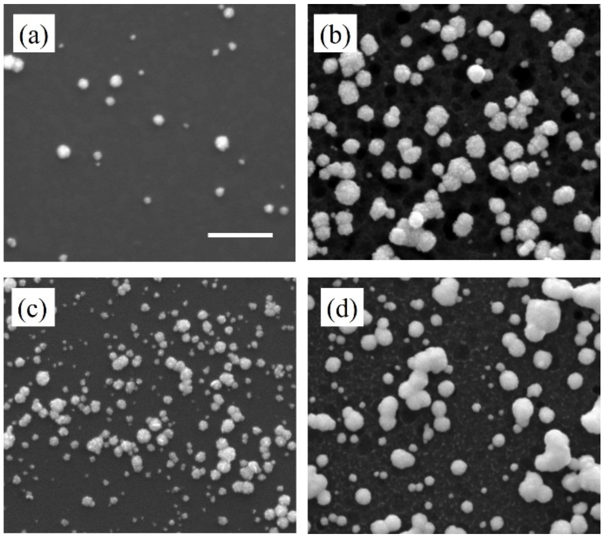

Figure S7. SEM images of Pt nanostructures deposited on Si substrate after (a) first, (b) tenth deposition cycle with SDS surfactant added, and (c) first, (d) tenth deposition cycle with CTAB surfactant added. The scale bar is 500 nm.

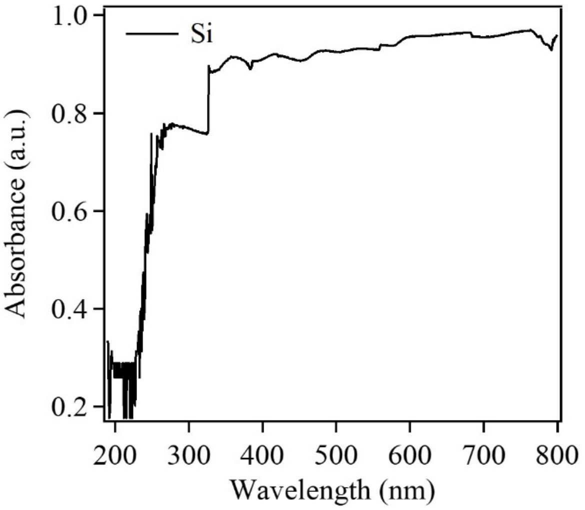

Figure S8. Normalized UV-Vis spectra of blank Si substrate.

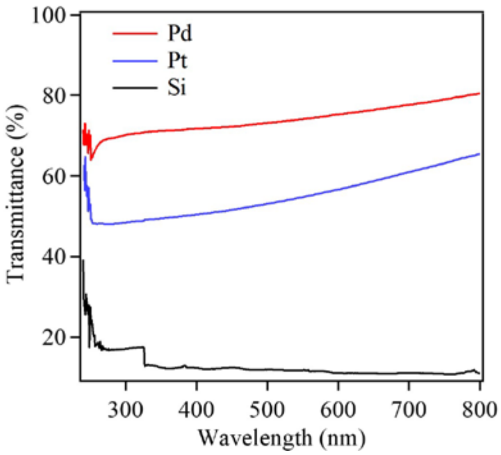

Figure S9: Transmittance data for Pd, Pt and Si.
